# Processing and Characterization of Unidirectional Flax Fiber-Reinforced Bio-Based Polyamide 11 Biocomposites

**DOI:** 10.3390/polym17050666

**Published:** 2025-02-28

**Authors:** Patrick Hirsch, Benjamin Tillner, André Henkel, Nico Teuscher, Ivonne Jahn, Maik Feldmann

**Affiliations:** Fraunhofer Institute for Microstructure of Materials and Systems IMWS, Walter-Hülse-Straße 1, 06120 Halle (Saale), Germany; benjamin.tillner@imws.fraunhofer.de (B.T.); ivonne.jahn@imws.fraunhofer.de (I.J.); maik.feldmann@imws.fraunhofer.de (M.F.)

**Keywords:** biocomposite, biopolymer, polyamide, flax fiber, UD tape

## Abstract

Within this study, the impregnation behavior and resulting mechanical properties of unidirectional flax fiber-reinforced polyamide 11 biocomposites were investigated. Therefore, different grades of bio-based polyamide 11 have been evaluated regarding their eligibility as composite matrix material. The production of the unidirectional flax fiber-reinforced biocomposites was investigated using a continuous film-stacking method. It was found that the flow behavior of the polyamide 11 matrix polymer significantly affected the impregnation quality and the resulting mechanical properties as tested by tensile and bending tests. A lower shear viscosity and stronger shear thinning behavior led to better impregnation, a 15% higher stiffness, and 18% higher strength. This was also analyzed with morphological analysis by scanning electron microscopy. Additionally, the effect of the fiber volume content of the flax fibers on the mechanical properties was tested, showing a positive correlation between the fiber content and the resulting stiffness and strength, leading to an increase of 48% and 55%, respectively. In result, a maximum Young’s modulus of 16.9 GPa and tensile strength of 175 MPa at a fiber volume content of 33% was achieved. Thus, the unidirectional flax fiber-reinforced polyamide 11 biocomposites investigated can be a sustainable construction material for lightweight applications, e.g., in the automotive industry.

## 1. Introduction

The market for biocomposites is driven by several factors. With increasing awareness of environmental issues, industries are seeking alternatives to reduce their carbon footprint and reliance on fossil fuels. Biocomposites offer a way to achieve this by utilizing renewable resources and reducing the use of non-renewable materials. Many regions are implementing stricter regulations and standards regarding the use of conventional plastics and non-sustainable materials [1]. Biocomposites can help companies comply with these regulations and demonstrate their commitment to sustainability and can supply this demand and appeal to environmentally conscious consumers. Research and development in material science have led to improvements in the mechanical properties, durability, and processability of biocomposites, making them more attractive for a wider range of applications [2]. Biocomposites are being adopted in industries such as automotive, construction, consumer goods, packaging, and more. For instance, natural fiber-reinforced plastics (NFRP) are being used in car interiors, furniture, and even aircraft components [2,3]. Governments and organizations in various countries are providing incentives and funding for the development and adoption of sustainable materials like biocomposites [1]. It is important to note that while the market for biocomposites is growing, there are still challenges to overcome, such as ensuring consistent quality, addressing supply chain issues for natural fibers, and improving the scalability of production processes [2,4,5,6].

However, as technology and awareness continue to progress, the market for biocomposites is likely to expand further. This is particularly true of thermoplastic biocomposites as they are also easy to recycle. Thus, thermoplastic biopolymers such as biobased polyamides and natural fibers such as cotton, hemp, flax, and others are relevant in various industries due to their unique properties and environmental considerations [7]. The high strength-to-weight ratio makes them a suitable choice of lightweight materials. Specifically, flax fibers provide significant strength and stiffness while being relatively lightweight with a density of 1.4 g/cm^3^ compared to traditional E-glass fibers with 2.54 g/cm^3^ [8]. When combined with bio-based polyamide 11 (PA11), which also offers good mechanical properties, the resulting biocomposites can have an excellent balance of strength and weight. PA11 exhibits properties such as high strength, flexibility, and resistance to chemicals, making it suitable for a wide range of applications, including automotive parts, consumer goods, medical devices, and more [9]. PA11 also provides additional flexibility, helping to prevent the composite from becoming too brittle. Both PA11 and flax fibers are bio-based and renewable materials that can be processed using techniques such as injection molding, compression molding, and even 3D printing [10,11,12]. This makes them versatile for various manufacturing processes. Combining them results in a composite that is more environmentally friendly compared to composites made from petroleum-based materials [11]. Flax fibers exhibit natural damping properties, which means they can absorb and dissipate energy from vibrations or impacts [2]. This can be beneficial in applications where vibration dampening is required, such as in automotive or aerospace components. Moreover, flax offers good thermal insulation properties, which can be advantageous in certain applications where temperature control is important [13]. Together, they create a biocomposite material that offers a compelling alternative to traditional lightweight materials while also addressing environmental and sustainability concerns [14]. However, scientists have also been studying how other natural fibers interact with PA11 as a matrix material and how they influence the mechanical strength, thermal stability, and other properties of the resulting biocomposites [12,14,15,16,17,18,19,20]. Additionally, other researchers have been developing efficient and scalable processing methods to manufacture biocomposites with consistent properties [7,21]. Studies have been conducted to assess the overall environmental impact of PA11-based biocomposites, considering factors such as raw material sourcing [22], manufacturing processes [23,24], use phase, and end-of-life options [25]. However, higher melting bio-based polyamides, such as polyamide 10.10 (PA10.10) or polyamide 6.10 (PA6.10), have also been studied as matrix polymers in biocomposites [26,27].

An important criterion for the resulting mechanical properties of biocomposites is the fiber length of the natural fibers used, with a higher fiber length leading to a higher level of mechanical properties. Unidirectional continuous fiber-reinforced thermoplastic tapes (UD tapes) represent a class of composite material with the highest potential in lightweight design in combination with high mechanical strength and stiffness. Depending on the used material systems (petrochemical or bio-based thermoplastics) and reinforcing fibers (glass, carbon, basalt, synthetic, or natural) different processing methods for the tape production can be used. The aim of the tape production is to impregnate the reinforcing fibers with the thermoplastic matrix polymer and reach the lowest possible void contents. According to the literature, different manufacturing methods for tape production based on powder, slurry, film, melt impregnation, or hybrid or commingled yarns are used [28,29]. For continuous reinforcing fibers, such as glass or carbon, the process of melt impregnation is the most efficient solution due to the use of fibers and thermoplastic granules coming directly from commercial suppliers. No preprocessing steps are requested for the melt or direct impregnation. In contrast, processes such as film stacking or commingled yarn consolidation are favored to produce thermoplastic tapes from continuous natural fibers [30]. The literature shows different manufacturing strategies for flax fiber-reinforced composites materials [31,32,33,34,35]. In addition to the use of unidirectional flax fabrics, there are processes either using discontinuous hot press molding or slurry-based fiber impregnation with additional consolidation steps. Also, hybrid or commingled yarns made of thermoplastic polymers and natural fibers have been used to improve the impregnation process.

However, only a few studies have been published on the production of continuously flax fiber-reinforced biocomposites with bio-based polyamides as matrix polymer as they have a comparatively high melting temperature and, therefore, very demanding process control in combination with natural fibers. Thus, this work focuses on the influences of the flow behavior of different bio-based PA11 types on the impregnation behavior during processing and resulting mechanical properties of continuous flax fiber-reinforced biocomposites.

## 2. Materials and Methods

### 2.1. Materials

Two different PA 11 Rilsan^®^ grades from Arkema^®^ (Düsseldorf, Germany) were combined with unidirectionally oriented flax fibers (FlaxTape^®^) from Ecotechnilin^®^ (Valliquerville, France). The dimensions of the flax tape were 400 mm in width and 0.25 mm in thickness. An overview of the material properties is given in Table 1.

### 2.2. Processing of Unidirectional Flax Fiber-Reinforced Polyamide 11 Biocomposites

**Figure 1:**
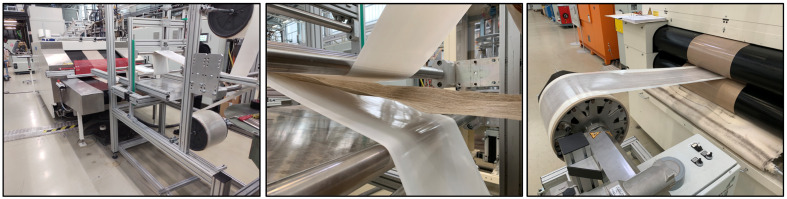
A processing line for PA11/flax tape production. The flax tape and film guiding system with a double-belt press (**left**), detailed view of the flax tape, and PA11 film before impregnation (**middle**) and winding of consolidated PA11/flax tape after impregnation (**right**).

The processing of unidirectional continuous flax fiber-reinforced biocomposites (UD tape) was carried out using a continuous film-stacking process. In the first step, PA 11 films were manufactured from granulate by extrusion using a sheet die. For this, an extruder from Fisher Scientific GmbH with a cast film die of 100 mm was used with a screw speed of 27 rpm and a constant barrel temperature of 210 °C. These pre-films have been subsequently pressed down to 160 ± 10 µm thickness to perform film stacking. Impregnation and consolidation were achieved using a double-belt press (Meyer GmbH, Salzgitter, Germany) in combination with a winder and a supply and guiding system for the non-woven flax tape combined with the PA11 films (see Figure 1). The impregnation of the flax tape with the continuously running press was performed under constant temperature of 220 °C and a speed of 1 m/min.

For mechanical testing, laminates of two different fiber volume fractions have been produced with the continuously running double-belt press. As a result, four different material combinations were prepared for further investigations, as shown in Table 2. To increase the fiber volume fraction, single layers of the already impregnated UD tape were stacked with additional layers of the flax tape (110 g/m^2^). These pre-stacked packages were again processed by the double-belt press to achieve consolidation of the different material layers. For later mechanical and morphological testing, specimens were cut out of the prepared laminates by mechanical cutting devices.

### 2.3. Rheological Characterization

In addition to the orientation and distribution of the reinforcing flax fibers, the performance of the biocomposite depends on the quality of the impregnation by the thermoplastic matrix. To investigate the influence of the used PA11 grades, rheological tests based on the melt flow index and shear viscosity were performed. The rheological characterization of the used PA 11 grades was carried out using high-pressure capillary viscometry according to ISO 11443. For this purpose, a capillary rheometer (Rheotester 2000) from Göttfert Werkstoff-Prüfmaschinen GmbH was used. In order to exclude any influence of absorbed water on the flow properties, the PA 11 materials were dried at 80 °C for 5 h before each experiment. Three round nozzles with a constant diameter of 1 mm and different lengths of 10, 20 and 30 mm were used for the tests. For each measurement, the apparent viscosities of PA 11 were determined at 10 different shear rates in the range from 3.9 to 2000 s^−1^ for each nozzle, with each measurement being repeated 3 times. The correction of the apparent viscosity determined was finally carried out according to Rabinowitsch–Weißenberg and Bagley. The Rabinowitsch–Weißenberg correction takes the real shear rate profile of structurally viscous polymer melts due to the wall adhesion into account, while the Bagley correction includes the viscoelastic inlet effects and the associated pressure drop in front of the nozzle in the calculation of the viscosity. Additionally, the melt flow index (MFI) according to ISO 1133 for melt temperatures of 200, 210, and 220 °C was measured for both PA11 polymers (Table 3). In result, the FMNO grade showed higher MFI values and, therefore, has a significantly lower viscosity and higher flowability as the BMNO grade.

### 2.4. Morphological Characterization

The morphology of the composite laminates was investigated before and after mechanical testing. Fiber distribution as well as consolidation quality were reviewed by cross-sectional light microscopic investigations using a microscope Olympus BX51 in bright field configuration with a magnification of 5. After mechanical testing, the surface of the broken composites was investigated by scanning electron microscopy (SEM) using a Quanta 3DFEG from FEI with 3 kV voltage and working distance of 10 mm. Thus, an Everhart–Thornley detector was used as the SEM detector. By using this method, more detailed information for fiber–matrix interaction and the interphase behavior under load could be determined. Additionally, investigations of the composite laminates by X-ray computer tomography (CT) were performed. The aim of the test was to identify the flax fiber orientation as well as the determination of the fiber volume fraction. Therefore, the consolidated multi-layer laminates were placed inside a Rayscan 200 E micro CT device, and a voltage of 90 kV and current of 200 µA were used for the scans. Each scan was performed with 1440 projections with 1750 ms time of integration and a voxcel size of 10 µm.

### 2.5. Mechanical Characterization

**Figure 2:**
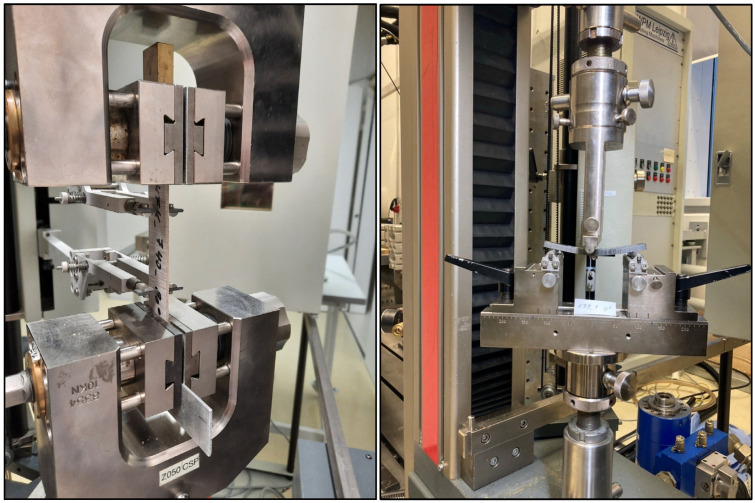
Test equipment for tensile test (**left**) and 3-point-bending test (**right**).

Mechanical investigations by tensile and 3-point bending tests were performed for the quantification of the effect of the impregnation quality of the unidirectional continuous flax fiber-reinforced biocomposites. For this, standard test specimen samples with a length of 150 mm and width of 20 mm were cut from the consolidated laminates by mechanical cutting according to the DIN ISO 527-5. For the 3-point bending test, specimens with 80 mm length and 15 mm width were prepared with a mechanical cutting device. The test specimens were pre-conditioned according to ISO 1110 at 70 °C and 62% relative humidity. The tensile properties in 0° and 90° in in terms of fiber orientation were determined according to DIN ISO 527-5 with a Zwick universal testing machine (Figure 2, left) with preload of 5 N and testing speed of 1 mm/min for the Young’s modulus and 5 mm/min for the tensile strength measurement. Further investigations of the composite bending behavior were carried out according to DIN 14125 with 3-point bending test using a Zwick testing device (Figure 2, right) with pre-tension of 0.1 MPa and measuring speed of 2 mm/min.

## 3. Results and Discussion

### 3.1. Rheological Behavior of Polyamide 11 Matrix Polymers

Physically, the process of fiber impregnation can be described as flow of a fluid through a porous media and can be modeled based on Darcy’s law [36,37,38]. Thus, the correlation between the impregnation flow q, the pressure gradient $\frac{dp}{dz}$, porosity K, and melt viscosity ƞ can be described as shown in Equation (1). For this model, the porosity of the fibers is assumed to be a constant average value and the pressure gradient will stay equal over the processing length. Therefore, the melt viscosity will be the parameter with the strongest effect on the impregnation quality.

(1) $$q=\frac{dL}{dt}=\frac{K}{ƞ}\frac{dp}{dz}$$

**Figure 3:**
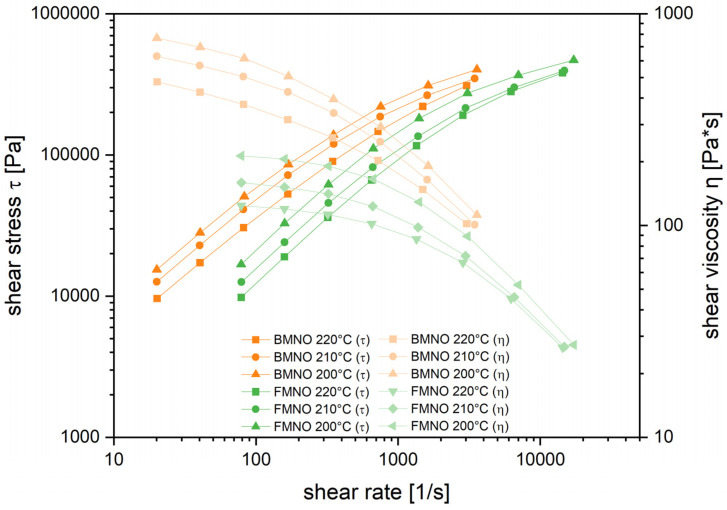
Shear stress and viscosity curves of the investigated PA11 grades at 200, 210, and 220 °C.

Based on the processing temperature range during the impregnation process, both PA11 grades were investigated by high-pressure capillary rheometer at 200, 210, and 220 °C. Figure 3 shows the temperature dependency for shear stress and shear viscosity in the shear rate region of 10 to 10,000 1/s. For both materials, the viscosity curves show a strong shear thinning behavior. In comparison to the BMNO, the FMNO grade has lower melt viscosity in the whole investigated shear rate range, which is assigned to a lower molecular weight. An increase in melt temperature resulted in a significant reduction in viscosity for both PA11 grades. This effect is of special interest for the processing of the unidirectional flax fiber-reinforced biocomposites with low shear rates using the double-belt press. According to Darcy’s law, the melt with lower viscosity will cause an increase in the impregnation flow and, therefore, improve the macro- and especially the micro-impregnation [39]. However, as shown by Kim and Lee [40], this can lead to a lower strength of the composite since a lower viscosity is attributed to a lower molecular weight.

**Figure 4:**
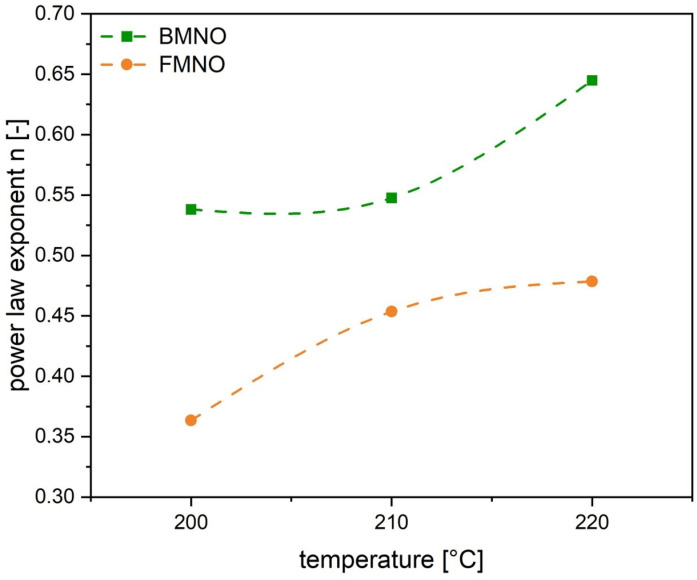
The effect of melt temperature on the power law exponent n of the investigated PA11 grades.

Based on the Carreau model (Equation (2)), the correlation of complex viscosity to zero shear viscosity, relaxation time, power law index and shear stress can be described [41]. The measured viscosity and shear stress curves were used for fitting the material parameters according to the model. The results, shown in Table 4, give valuable material information for the shear thinning behavior for both PA11 grades. For both polymers, the zero shear viscosity as well as the power law exponent show a significant temperature dependency. A reduced power law exponent is an expression for an increased shear thinning behavior and, therefore, improved impregnation of the reinforcing fibers [42]. Comparing the power law exponents in Figure 4, it is obvious that the FMNO has the lower power law exponent compared to the BMNO. Thus, in combination with the lower zero shear viscosity, the FMNO is considered more suitable for low shear rate impregnation processes such as the film stacking process used for the processing of the unidirectional flax fiber-reinforced biocomposites in this study.

(2) $$\eta =(\eta 0-\eta \infty )/({\left[1+{\left(\lambda \cdot \dot{\gamma }\right)}^{c}\right]}^{\frac{1-n}{c}})$$

### 3.2. Impregnation Quality of Unidirectional Flax Fiber-Reinforced Polyamide 11 Biocomposites

**Figure 5:**
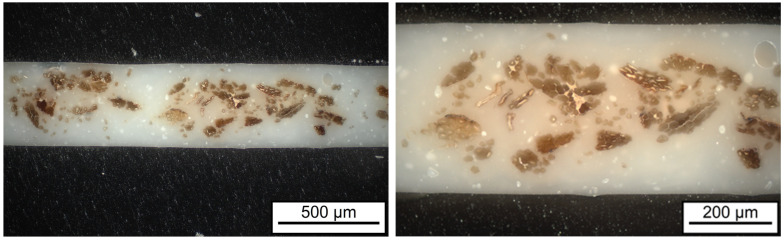
Impregnation quality of FMNO/FT20 as shown by light microscopy.

In addition to the fiber type, the fiber content, the chemical composition, the fiber orientation, and the impregnation quality, in particular, influence the resulting mechanical properties of natural fiber-reinforced composite materials. Figure 5 shows the composite material morphology of the material FMNO/FT20 obtained by light microscopy. It shows both partially strongly agglomerated fiber bundles and pronounced skin layers with a high proportion of matrix polymer. It can also be seen that there are no voids between the flax fibers and the matrix polymer. From this, it can be concluded that the film impregnation process used has led to a sufficiently good impregnation quality. However, from the analysis, it is also obvious that the fiber content could be increased. In addition, improved spreading of the fibers should lead to a more homogeneous distribution within the UD tape. Overall, these results demonstrate the technical feasibility of producing UD tapes from bio-based PA11 and flax fibers using film impregnation with a sufficiently good impregnation quality. This was already stated by Le Duigou et al. [43], who investigated the interfacial properties throughout the melt processing of biocomposites from PA11 and flax fibers. It was found that the practical adhesion is mainly governed by van der Waals and hydrogen interactions rather than radial residual thermal stresses.

**Figure 6:**
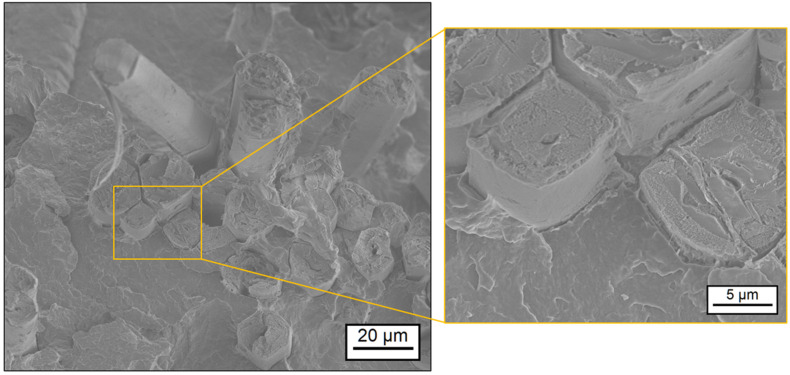
Impregnation quality of FMNO/FT20 as shown by scanning electron microscopy.

**Figure 7:**
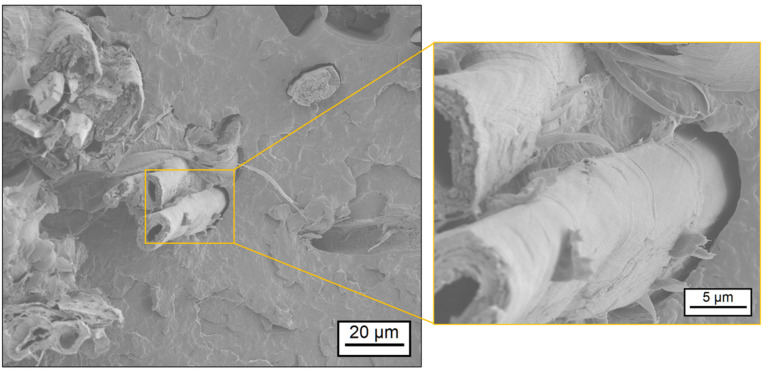
Impregnation quality of BMNO/FT20 as shown by scanning electron microscopy.

Using scanning electron microscopy (SEM), the correlation between the rheological properties of the matrix polymer and the resulting impregnation quality was studied. Thus, the surface of fractured specimen of the composites was investigated with different magnitudes. Figure 6 and Figure 7 show sections of the specimen’s surface with a detailed view on fiber–matrix bonding areas. The composite FMNO/FT20 in Figure 6 shows good bonding between the flax fibers and the embedding matrix. The fibers are densely surrounded by the FMNO matrix polymer, and only small voids between fiber and matrix can be found. In contrast, the composite BMNO/FT20 in Figure 7 shows more voids between the fibers and the matrix polymer. Furthermore, the fiber structure seems to be not completely infiltrated by the BMNO matrix, which shows reduced micro-impregnation. From this finding, a correlation between the shear thinning behavior of the PA 11 matrix polymer and the resulting impregnation quality of the flax fibers can be concluded. The lower melt viscosity and the higher shear thinning behavior of the FMNO leads to better macro- and micro-impregnation of the flax fibers compared to the BMNO. Complete impregnation and consolidation of the composite is necessary to reach the highest possible performance in continuous natural fiber-reinforced composites [29]. In case of void formation during impregnation and consolidation, the load transfer efficiency between matrix and fiber is less, causing a decrease in mechanical performance of the composite system [44].

**Figure 8:**
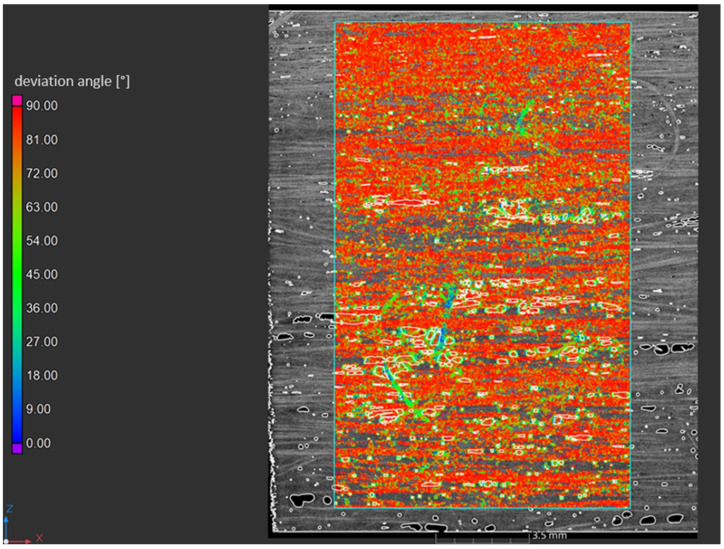
Main fiber orientation angle of FMNO/FT20 as shown by computer tomography.

The main fiber orientation angle of the biocomposites was investigated by CT scans. Based on this study, the orientation angle of visible fibers was determined and the deviation from the main angle was analyzed. In Figure 8, the scanned and analyzed fiber orientation angle of the FMNO/FT20 biocomposite is shown. On the right side, the analyzed fiber orientation distribution is depicted as a histogram. Thus, the majority of the red colored flax fibers indicate that the main fiber orientation is aligned to the main orientation of the UD tape.

### 3.3. Mechanical Properties of Unidirectional Flax Fiber-Reinforced Polyamide 11 Biocomposites

**Figure 9:**
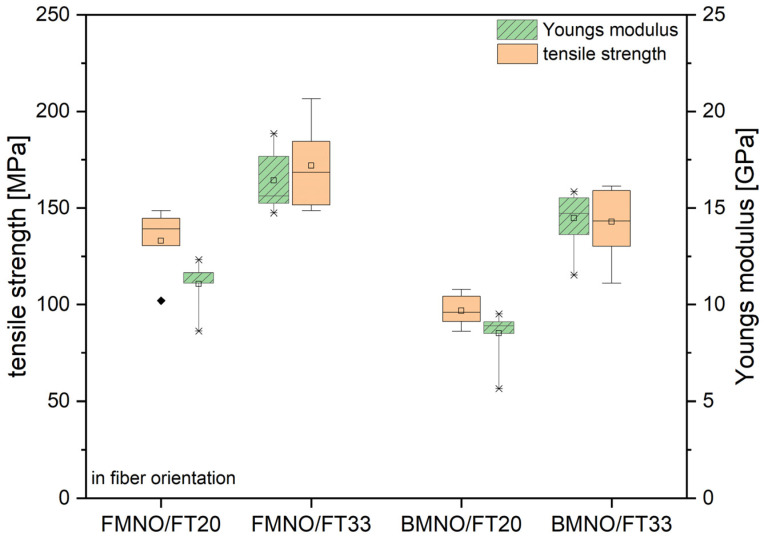
Mechanical properties of the FMNO/FT and BMNO/FT biocomposites as measured by the tensile test in fiber orientation after conditioning according to ISO 1110.

Mechanical characterization of the four unidirectional flax fiber-reinforced biocomposites was carried out by tensile tests along and across to the fiber orientation as well as by 3-point bending tests after conditioning according to ISO 1110. As can be seen in Figure 9 and Table 5, the tensile strength and Young’s modulus show a strong correlation between the fiber content and the mechanical properties. The increase in the natural fiber volume content from 20 vol% to 33 vol% caused an increase in the tensile strength and Young’s modulus of 55% and 48%, respectively. Comparing both matrix polymers with respect to their effect on the composite properties, it was found that the FMNO-based composite shows higher stiffness and strength as measured by the tensile test. In specific, the Young’s modulus and tensile strength of the FMNO composite with a fiber volume content of 33% were measured as 16.9 GPa and 175 MPa along the fiber orientation, respectively. For the same fiber volume content, the Young’s modulus and tensile strength of the BMNO composite were measured as 14.5 GPa and 143 MPa. In comparison, this represents a 15% lower stiffness and 18% lower strength. This is attributed to a higher impregnation quality as already concluded from the rheological and morphological characterization. However, these values are in good correlation with mechanical properties of other flax fiber-reinforced composites reported by Yan et al. [45]. Sarkar et al. [24] investigated the use of flax fabrics in combination with different thermoset matrices. Comparing this thermoset and the thermoplastic variant based on PA11 in this study, the FMNO/FT33 shows higher tensile properties in fiber orientation compared to the epoxy/flax biocomposite with 30 vol% fiber content in the study of Sarkar et al. [24]. However, tensile properties of biocomposites from PA11 and flax fibers reported by Lebaupin et al. [7] were significantly higher. They achieved a Young’s modulus of 36 GPa and a tensile strength of 174 MPa at a fiber weight content of 50% but with significant porosities up to 26%.

**Figure 10:**
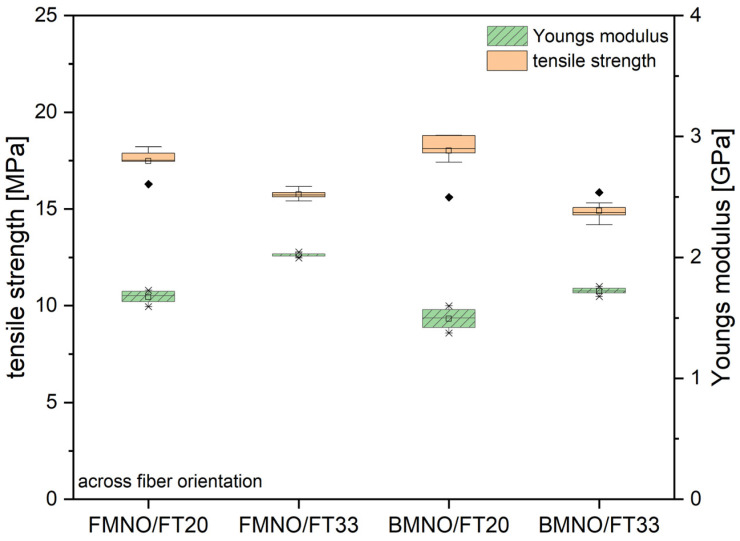
Mechanical properties of the FMNO/FT and BMNO/FT biocomposites as measured by the tensile test across fiber orientation after conditioning according to ISO 1110.

Testing of the tensile properties of the unidirectional flax fiber-reinforced biocomposites across the fiber orientation showed strong anisotropic behavior. This can be seen in Figure 10 where the tensile strength and Young’s modulus are depicted for the four different material combinations depending on their fiber volume fraction. The highest value for the Young’s modulus was measured as 2.1 GPa for the FMNO/FT33 composite. Interestingly, the tensile strength was found to be comparable for the FMNO/FT20 and BMNO/FT20 biocomposites with a value of 18 MPa. Increasing the fiber content led to a decrease in both composites to 14.1 MPa and 14.9 MPa for the FMNO/FT33 and BMNO/FT33, respectively.

**Figure 11:**
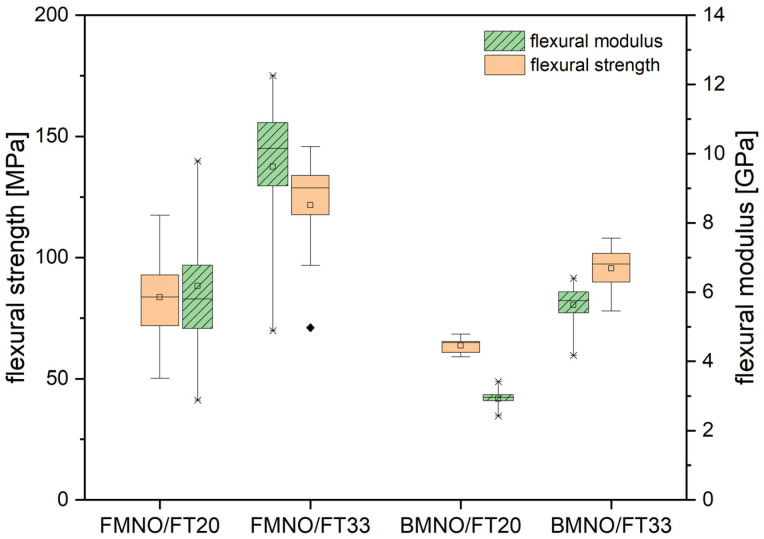
Mechanical properties of the FMNO/FT and BMNO/FT biocomposites as measured by 3-point bending test after conditioning according to ISO 1110.

Three-point-bending tests were carried out according to DIN 14125, and after conditioning, according to ISO 1110. The resulting flexural strength and modulus are depicted in Figure 11 as measured with a testing speed of 2 mm/min. In good correlation with the results from the tensile test, the results of the bending test show a significant matrix influence on fiber orientation. The composites processed with the FMNO grade show higher strength and stiffness for both investigated fiber contents. Increasing the fiber volume content from 20 to 33 vol% results in a flexural strength of 127 MPa and a corresponding bending modulus of 9 GPa. Comparing this result with the epoxy/flax tape reference from Sarkar et al. [24] in Table 5, it is obvious that the higher matrix stiffness of the epoxy resin increases the composites flexural behavior above the thermoplastic material system. However, as long as there is no industrially feasible solution for recycling thermoset fiber composites, thermoplastic fiber composites have a major advantage in terms of recycling and sustainability. The biocomposites examined in this study are, therefore, of great interest for lightweight construction applications, especially in the automotive industry, e.g., for sustainable seat structures [46,47].

## 4. Conclusions

In summary, biocomposites are relevant due to their potential to address environmental concerns and sustainability issues associated with traditional plastics and synthetic fibers. In this study, the processing and resulting mechanical properties of unidirectional flax fiber-reinforced polyamide 11 biocomposites were investigated. The following conclusions have been drawn based on the findings of the study:

- The rheological properties of the PA 11 matrix polymer, in particular the shear thinning behavior, have a significant influence on the resulting impregnation quality. A lower viscosity and a stronger shear thinning behavior as described by a lower power law index is preferable for better macro- and micro-impregnation of the flax fibers.
- An improved impregnation quality leads to increased mechanical characteristics, as demonstrated for the FMNO/FT biocomposites examined based on the higher Young’s modulus and tensile strength for both fiber contents of 20 and 33% investigated.
- The mechanical properties of unidirectional flax fiber-reinforced polyamide 11 biocomposites are comparable to epoxy-based flax UD tapes. Thus, the biocomposites investigated represent a more sustainable and cost-effective alternative as they are recyclable and efficiently processable. This results in a wide range of sustainable application possibilities, such as lightweight automotive parts using thermoforming or hybrid injection molding processes.

## Figures and Tables

**Table 1:** Overview of the properties of the used raw materials.

Property	Unit	Rilsan^®^ BMNO	Rilsan^®^ FMNO	FlaxTape^®^
Density	g/cm^3^	1.03	1.03	1.40
Areal weight	g/m^2^	165.00	165.00	110.00
MFI (220 °C, 2.16 kg)	g/10 min	20.17	57.84	-
Melting temperature	°C	189.00	189.00	-
Young’s modulus	GPa	1.28	1.20	53.20 ± 12.70
Tensile strength	MPa	41.00	39.00	1045.00 ± 273.00
Water absorption	%	1.90	0.57	8.00–12.00

**Table 2:** Overview and denomination of the processed unidirectional flax fiber-reinforced biocomposites.

Material ID	PA11 Grade	Fiber Volume Fraction (%)	Thickness (mm)	Number of Layers	
				Bio-UD-Tape	Flax-Tape
FMNO/FT20	FMNO	20 ± 2	2 ± 0.1	5	0
FMNO/FT33	FMNO	33 ± 2	2 ± 0.1	4	4
BMNO/FT20	BMNO	20 ± 2	2 ± 0.1	5	0
BMNO/FT33	BMNO	33 ± 2	2 ± 0.1	4	4

**Table 3:** Determined MFI values for the investigated PA11 grades at 200, 210 and 220 °C.

Parameter	Unit	FMNO			BMNO		
Temperature	°C	200	210	220	200	210	220
Weight	kg	2.16	2.16	2.16	2.16	2.16	2.16
MFI	g/10 min	32.26	44.10	57.84	14.17	17.13	20.17

**Table 4:** Converged fitting parameters for the Carreau model with R^2^ = 0.99 from high-pressure capillary rheometer measurements of the PA11 grades according to ISO 11443.

Parameter	Unit	Description	FMNO			BMNO		
T	°C	Temperature	200	210	220	200	210	220
*η*∞	Pa·s	Infinite shear viscosity	0.00	0.00	0.00	0.00	0.00	0.00
*η*0	Pa·s	Zero shear viscosity	213.64	158.64	122.71	780.20	632.74	492.60
*λ*	s	Critical shear rate	0.01	0.01	0.02	0.00	0.00	0.00
n	-	Power law exponent	0.36	0.45	0.48	0.54	0.55	0.64
c	-	Transition parameter	1.50	1.50	1.50	1.50	1.50	1.50

**Table 5:** Mechanical properties of the FMNO/FT and BMNO/FT biocomposites as measured by tensile test and 3-point-bending test in comparison to an epoxy/flax tape [24].

Test Method	Test Parameter	Unit	FMNO/FT		BMNO/FT		Epoxy/Flax [24]
Analytic Calculation	Fiber content	Vol%	20	33	20	33	30
Tensile Test (in fiber orientation)	Young’s modulus	GPa	11.4	16.9	8.5	14.5	7.1
	Tensile strength	MPa	112.9	175.5	96.8	143.0	115.8
Tensile Test (across fiber orientation)	Young’s modulus	GPa	2.0	2.1	1.5	1.7	-
	Tensile strength	MPa	18.1	14.1	18.0	14.9	-
3-Point-Bending Test	Flexural modulus	GPa	6.2	9.6	2.9	5.6	9.4
	Flexural strength	MPa	83.6	122	63.7	95.4	130.9

## Data Availability

Data are available upon request from the authors.
